# What constitutes equitable data sharing in global health research? A scoping review of the literature on low-income and middle-income country stakeholders’ perspectives

**DOI:** 10.1136/bmjgh-2022-010157

**Published:** 2023-03-28

**Authors:** Natalia Evertsz, Susan Bull, Bridget Pratt

**Affiliations:** 1 Royal Melbourne Hospital, Melbourne, Victoria, Australia; 2 Nuffield Department of Population Health, University of Oxford, Oxford, UK; 3 Department of Psychological Medicine, The University of Auckland, Auckland, New Zealand; 4 Queensland Bioethics Centre, Australian Catholic University, Banyo, Queensland, Australia

**Keywords:** Health policies and all other topics, Review

## Abstract

**Introduction:**

Despite growing consensus on the need for equitable data sharing, there has been very limited discussion about what this should entail in practice. As a matter of procedural fairness and epistemic justice, the perspectives of low-income and middle-income country (LMIC) stakeholders must inform concepts of equitable health research data sharing. This paper investigates published perspectives in relation to how equitable data sharing in global health research should be understood.

**Methods:**

We undertook a scoping review (2015 onwards) of the literature on LMIC stakeholders’ experiences and perspectives of data sharing in global health research and thematically analysed the 26 articles included in the review.

**Results:**

We report LMIC stakeholders’ published views on how current data sharing mandates may exacerbate inequities, what structural changes are required in order to create an environment conducive to equitable data sharing and what should comprise equitable data sharing in global health research.

**Conclusions:**

In light of our findings, we conclude that data sharing under existing mandates to share data (with minimal restrictions) risks perpetuating a neocolonial dynamic. To achieve equitable data sharing, adopting best practices in data sharing is necessary but insufficient. Structural inequalities in global health research must also be addressed. It is thus imperative that the structural changes needed to ensure equitable data sharing are incorporated into the broader dialogue on global health research.

**WHAT IS ALREADY KNOWN ON THIS TOPIC**

- Data sharing is thought to be an ethical imperative due to its potential to maximise the value and utility of collected data.
- There is growing consensus that data sharing should be equitable; however, there is still no clear concept of equitable data sharing.

**WHAT THIS STUDY ADDS**

- As a matter of procedural fairness and epistemic justice, the perspectives of low-income and middle-income country stakeholders must inform concepts of equitable health research data sharing.
- This paper investigates their recent views in relation to how equitable data sharing in global health research should be understood.

**HOW THIS STUDY MIGHT AFFECT RESEARCH, PRACTICE OR POLICY**

- This review found that implementing best-practices in equitable data sharing is insufficient on its own.
- Structural change is required in order for equitable data sharing to be possible.

## Introduction

Data sharing is increasingly viewed as an important component of good science, due to its role in promoting research integrity, enabling review of research findings, increasing the statistical power of meta-analyses and making efficient use of resources.^1^ Arguments that data sharing is an ethical imperative rest on its potential to maximise the value and utility of collected data, limiting unnecessary duplication of research, and thereby reducing the burdens and potential harms to research participants.^2–4^ It is generally assumed that data sharing will play an important role in improving health outcomes.^5–11^

This paper focuses on the sharing of primary research datasets and metadata generated during health-related research in low-income and middle-income countries (LMICs). In LMICs, relatively limited research resources have resulted in disproportionately smaller amounts of data being collected relative to disease burdens.^12^ Sharing research data collected from LMICs is expected to make significant contributions to global health by maximising the available evidence to inform healthcare, health policy, health system funding and regulatory decisions.^2–4 13 14^ Thus, data sharing is increasingly mandated by journals, publishers and funders, and calls have been made to strengthen the current mandates, requiring scientists worldwide to commit research data to publicly accessible databases within specified timeframes.^15^ Powerful public and philanthropic stakeholders have profoundly influenced the development of international standards for such sharing and institutional policies requiring data sharing as a condition of research support.^5 10 16^

Despite the potential advantages of sharing health research data to maximise secondary analyses, such sharing raises several challenges related to equity and justice. Where the curation and sharing of data is not meaningfully recognised or rewarded, it is likely that the benefits of data sharing will primarily accrue among researchers conducting and publishing secondary analyses.^17 18^ In such contexts, data sharing mandates may exacerbate inequities between researchers conducting primary research and sharing data, and researchers accessing that data and conducting secondary analyses. Due to structural constraints, primary researchers in LMICs can become entrenched in the role of data production, a role which is not meaningfully rewarded under the current system. Meanwhile, secondary researchers in high-income countries (HICs) analyse and publish findings and are rewarded with further funding and career opportunities.^19–21^

In recognition of these challenges, in 2011, the major funders of global health research jointly called for public health data to be shared in ways that are equitable, ethical and efficient.^22^ Yet, in 2022, a clear concept of equitable research data sharing has still not been defined.^23^ This presents a key challenge to developing policies and processes for equitable data sharing in global health research. There is a need for a clear and rigorous concept of equitable data sharing that reflects and incorporates the voices of all stakeholders, including LMIC researchers and communities.

In this paper, we investigate LMIC stakeholders’ published views in relation to what comprises equitable data sharing in global health research. Building on the work of Bull *et al*,^1^ we conducted a scoping review of the most recent 6 years of published literature on LMIC stakeholders’ experiences and perspectives of data sharing in global health research and thematically analysed the identified literature. We report views on how current data sharing practices may exacerbate inequities, what benefits accrue from equitable data sharing, what barriers exist to equitable data sharing, what foundations are required to create an environment conducive to equitable data sharing and what comprises equitable data sharing in global health research. In light of our findings, we conclude that achieving equitable data sharing requires not only adopting best practices in data sharing but also addressing structural inequalities in global health research.

## Methods

Scoping reviews seek to identify literature relevant to the research objective and may include a variety of article formats.^24 25^ This scoping review sought to identify a published literature on LMIC stakeholders’ experiences and perspectives of data sharing in global health research from 2015 onwards. An initial search strategy using a combination of subject headings and text words was developed in consultation with a reference librarian and run through MEDLINE (OvidSP). Papers discussing data sharing issues that specifically mentioned equity/justice/marginalisation and LMICs or global health in the abstract were identified. The keywords from these articles were analysed and incorporated into a revised search strategy, along with keywords from relevant articles. The revised search strategy was then finalised with the librarian (see online supplemental file 1). On the 23 September 2020, the following databases were searched: Embase (OvidSP), Global Health (OvidSP), Global Health (CAB Direct) and Web of Science Core Collection (Clarivate Analytics). The search was limited to studies about humans reported in English and published between 2015 and 23 September 2020.

10.1136/bmjgh-2022-010157.supp1 Supplementary data

**Figure 1:**
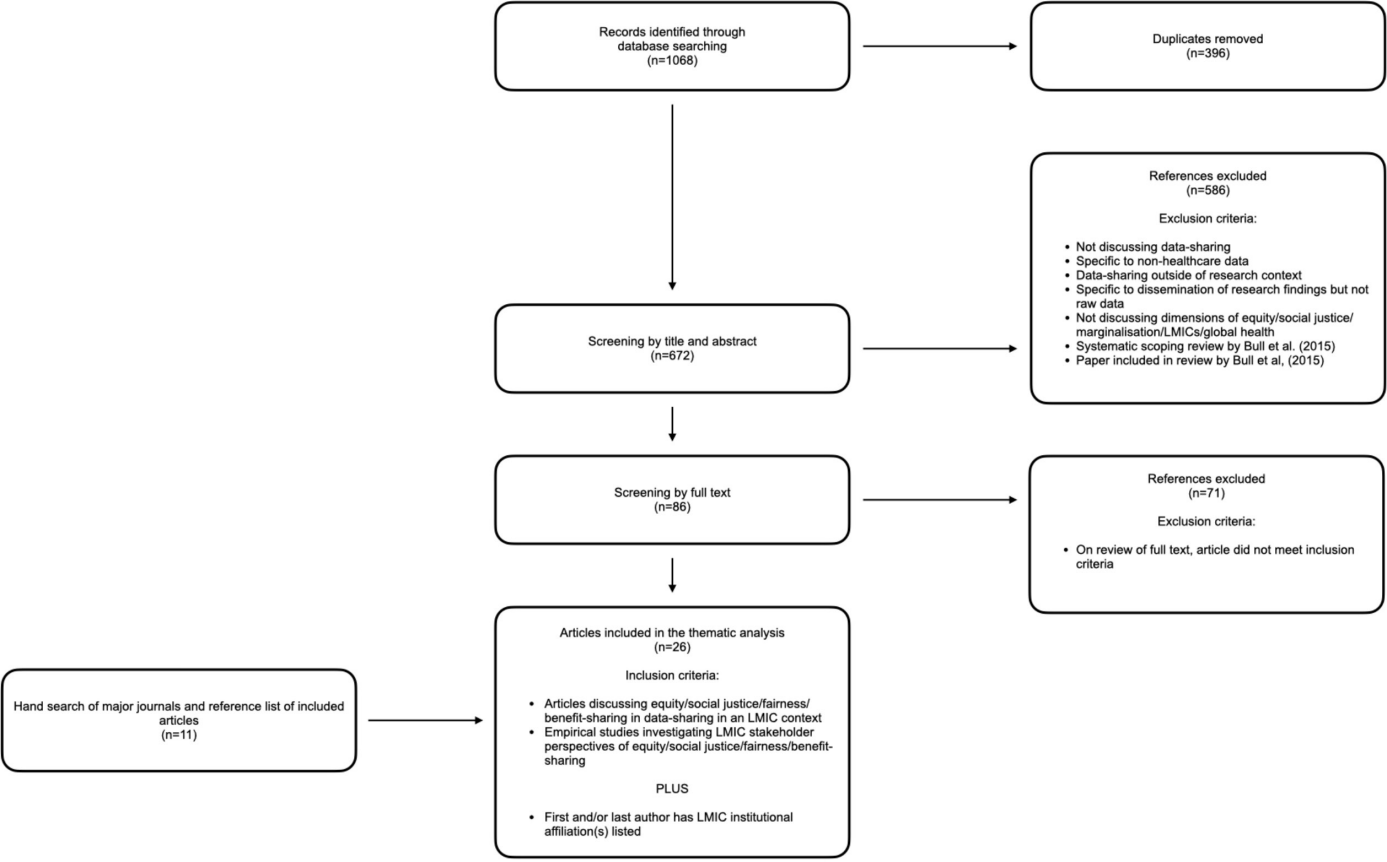
Preferred Reporting Items for Systematic Reviews and Meta-Analyses diagram. LMICs, low-income and middle-income countries.

A total of 1068 references were returned and imported into Covidence, which removed 396 duplicates. The remaining 672 articles were screened by title and abstract according to a matrix of inclusion and exclusion criteria (figure 1). The remaining 86 articles were screened by full text and 15 met the inclusion criteria. A hand search of the included literature identified a further 11 articles. All abstracts and full texts were reviewed by the first author. All full-text articles that were considered ‘possible inclusions’ by the first author were also reviewed by the last author and a mutual decision made on their inclusion.

Thematic analysis was performed on the included 26 articles in the following five stages: familiarisation with the data, initial coding framework creation, coding, finalisation of the coding framework and recoding of articles for consistency with the final framework.^26–28^ Given the limited pre-existing knowledge of LMIC stakeholders’ views on equitable data sharing, an inductive approach to coding was used. An inductive approach allowed us to code the data in a way that was responsive to stakeholders’ views, rather than seeking to fit them into an existing framework. The coding framework was coreviewed by the first and last authors before it was finalised. Coding of full-text articles was then performed by the first author using NVivo V.1.5. The five emergent themes identified were: threats to equity from unfair data sharing, benefits of equitable data sharing, barriers to equitable data sharing, foundations for equitable data sharing and features of best practice in equitable data sharing.

### Authors’ positionality

As in all research, it is important to understand our positionality and, therefore, how our professional and life experiences informed our understanding of the data. The first author conducted the search, coding and analysis of the literature. She is a medical doctor from Australia, with 4 years of prior experience working on empirical ethics projects related to global health research. Those projects raised themes of power, colonial relations and structural barriers to achieving equity.

The second author is a bioethicist currently based in New Zealand, whose research interests have focused on global health ethics since 1999. Over the past decade, she has conducted empirical ethics research on data and sample sharing and biobanking in collaboration with colleagues in Australia, Ghana, Hong Kong, India, Kenya, Malawi, South Africa, Thailand, UK and Vietnam.^1 19–21 29–40^ She has a longstanding interest in inequities and the exercise of power during the construction of global health data sharing landscapes and associated knowledge production processes.

The study was supervised by the last author, a bioethicist who grew up in the USA and has lived most of her adult life in Australia. She has over 10 years’ experience conducting global health ethics research with HIC and LMIC collaborators. This includes qualitative studies in Uganda, India, Thailand and the Philippines.^41–44^ In conducting her research, she is immersed in studying concepts and issues of equity, power, epistemic justice, social justice and global justice.

Our expertise and experience led us to see the data through a structural lens, which is reflected in our conclusions. However, we also undertook a reflexive approach to data analysis and made a conscious effort to be aware of our positionalities and remain grounded in the data when interpreting the results and the implications of the study. None of us are from LMICs, but we are committed to reducing epistemic injustice within global health.^45 46^ We undertook this study to highlight LMIC voices in relation to data sharing, not our own HIC voices, and that aim guided us throughout.

### Patient and public involvement

This scoping review was conducted as a student research project at the University of Melbourne. No primary data was collected as part of this project and it was not appropriate or possible to involve patients or the public in the design, or conduct, or reporting, or dissemination plans.

## Results

### Literature reviewed

Of the 26 articles, 10 reported the results of qualitative studies of LMIC stakeholders’ perspectives on ethical data sharing; 1 reported a quantitative survey; 9 were editorials, commentaries or conceptual analyses; and 6 documented case studies of data sharing (online supplemental file 2). The 17 empirical studies collected views of senior and junior researchers, fieldworkers, research participants, community representatives, members of research ethics committees and members of local regulators and government.^19–21 31 33 34 47–51^ The case studies were based in Argentina, Guatemala, the Dominican Republic, India, Pakistan, Sri Lanka, Thailand, Kenya, Liberia, Sierra Leone, South Africa and Zambia (Some papers featured a number of case studies from different geographical regions.^52 53^), in addition to 1 that focused on the Worldwide Anti-Malarial Resistance Network, which comprises over 280 global collaborators.^52–57^ Of the 26 articles included, 16 were from institutions or projects associated with the Wellcome Trust.^12 19–21 31 33 34 49 52–55 57–60^ In 23 out of the 26 papers, at least 1 author had an HIC institutional affiliation, which potentially reinforces concerns about the dominance of HIC stakeholder perspectives in global health data sharing landscapes. None of the reviewed papers were exclusively authored by researchers with HIC institutional affiliations, although in some cases either the first or last author had such an affiliation.

10.1136/bmjgh-2022-010157.supp2 Supplementary data

### Concerns about inequity in data sharing

The identified literature described numerous threats to equity that arise due to unfair data sharing (table 1).

**Table 1:** Summary of potential benefits and threats to equity from data sharing

Advancing equity through fair data sharing	Threats to equity from unfair data sharing
Benefits to LMIC researchers Researchers receive fair rewards for collection and sharing of data Fostering equitable collaborations Increased publications, leading to reputational/career benefits and further funding opportunities	Threats to LMIC researchers Worsening of unfair power relations with HIC researchers Opportunity and fiscal cost unfairly burden poorly resourced researchers and institutions; and the rewards are insufficient to offset the burdens Exclusion from participation in advancing science (LMIC researchers are stuck in a cycle of data production only) Career and reputational damage leading to loss of funding opportunities Lack of recognition hampers career progression
Benefits to LMIC participants and communities Secondary research is actualised to local or national public health benefit, leading to improved individual and community health and reduction of the global inequality in disease burdens Reduced duplication of research reduces the burden of repeated participation in studies on marginalised participants Education and empowerment of participants during the consent process and inclusion in the creation of data sharing policies	Threats to LMIC participants and communities Commercial exploitation Stigmatisation Benefits of research are not shared with originating communities
Benefits to local health research systems Capacity building of local researchers and research institutions	Threats to local health research systems Reduced opportunity to develop local research infrastructure and personnel

HIC, high-income country; LMIC, low-income and middle-income country.

Data sharing was described as unfair when it potentially exacerbated inequities between HIC and LMIC researchers, between LMIC research participants and communities and HIC populations and between HIC and LMIC health research systems. The most frequently raised concern centred around the capacity of unfair data sharing practices to reinforce unequal power relations and neocolonial behaviours, where HIC researchers mined data from LMICs without sharing the benefits with LMIC researchers or populations.^12 31 47 49–53 60–63^ Global inequities in the research enterprise and knowledge production mean that many LMIC researchers do not have the time, human or financial resources to devote to secondary analyses. Consequently, the benefits of such sharing will primarily accrue to HIC analysts and institutions in the form of future funding opportunities, career progression and reputation, thereby further compromising opportunities to develop LMIC research infrastructure and personnel.^20 31 62^ Without such opportunities, disparities between HIC and LMIC research systems will remain. Concerns were also expressed that unfair data sharing practices risk widening the research-output gap between HIC and LMIC researchers, perpetuating a dynamic where LMIC researchers are entrenched in the role of data production while HIC researchers analyse and publish findings.^19–21^ The costs of data sharing were also thought to unfairly burden poorly resourced researchers and institutions without returning commensurate benefits, furthering exacerbating inequities between LMIC and HIC researchers.^21 31 49 50 52 53 61 62^

Where secondary researchers are removed from the context of the primary research, it was noted that secondary analyses can result in inaccurate conclusions and stereotyped reporting, thus undermining the integrity of the primary research and interrupting its translation to health outcomes in LMICs.^20 52 59 63^ Concerns were also raised that where translational benefits to health outcomes accrued from secondary analysis, they will not be made available in the LMIC communities in which the data were collected.^34 53 64^ These factors were ultimately thought to obstruct the equitable distribution of benefits to originating communities that would otherwise be derived from data sharing.^53^

### Views on benefits of equitable data sharing

The identified literature described numerous benefits of equitable data sharing in global health research (table 1), which, in turn, advance equity between HIC and LMIC researchers, between LMIC populations and HIC populations and between HIC and LMIC health research systems. Key potential benefits of equitable data sharing for LMIC researchers related to capacity, collaboration and recognition. They included fair rewards for LMIC researchers’ efforts and equal opportunities for career development, including increased publications and research funding.^31 61^ Where systemic barriers obstructed capacities to access and analyse shared data (eg, limited analytic capacity due to scarce technological resources, lack of trained staff and funding), their removal was also linked to enhanced productivity and competitiveness of LMIC scientists. This, in turn, can ultimately help to reduce global disparities in research output.^53 61^ Collaborative partnerships between data sharers and data accessors were seen to have a number of benefits for LMIC researchers, including increased opportunities for authorship, skill and knowledge sharing, upskilling in data sharing and improvements to the quality and rigour of research.^19 31 47^ This was thought to reduce the global divergence in research outputs and activities.^48^ Potential benefits of equitable sharing to LMIC participants and communities rested on the translation of secondary research into local or national public health benefits,^19 31 34 52 59^ which can help reduce health disparities between LMIC and HIC populations.

### Views on barriers to equitable data sharing

Numerous environmental, personal and relational barriers to equitable data sharing were discussed (table 2).

**Table 2:** Summary of barriers to and foundations of equitable data sharing

Barriers preventing equitable data sharing	Foundations of equitable data sharing
Environmental barriers: Background structural conditions preventing equitable data sharing Unequal research systems, resources and capacity between HICs and LMICs Lack of fiscal resources for primary research and data curation and sharing Limited local policy or legal frameworks to govern and enforce measures to make data sharing equitable Lack of enforceability of data sharing agreements and policies Competitive scientific research environment, and rewards grounded in publication Relational and personal barriers Fear of imbalance between burdens and reward Experience in data sharing Trust between primary researchers and secondary researchers Concerns surrounding data sovereignty	Environmental foundations: Creating robust governance structures Establish frameworks and policies that support equitable data sharing, backed by enforceable governance mechanisms Governance mechanisms should be: Fair, ethical and accountable Implemented at all levels: from institutional to international levels Harmonised across levels to ensure equitable relations across borders Developed in consultation with community members Flexible to account for technology advances Accountable and subject to external review Redressing inequities in capacity Capacity building of LMIC research systems; provision of resources for sustainable and long-term data sharing Relational foundations: Building trust Alignment of interests between primary and secondary researchers Institutional policies supporting equitable data sharing Long-term relationships and collaborative partnerships with secondary researchers

HICs, high-income countries; LMICs, low-income and middle-income countries.

Environmental barriers, which comprise the structural conditions that prevent equitable data sharing, were the most prominent theme in the literature. All identified sources described a lack of resources and capacity as a barrier to equitable data sharing. Many researchers conduct research in their personal time, aided by personal finances, leaving little time or money for data sharing.^47 48 50 50 61^ Lack of secure access to the internet and computers, inability to access library resources off-site, insufficient funds for software and the professional memberships and subscriptions required for effective participation in Open Science, and poor digital and data science literacy exclude LMIC researchers from meaningfully participating in data sharing.^31 48 51 61 63^ A lack of core infrastructure funding from institutions, governments and funders means researchers have little way to tackle these barriers.^50^ Furthermore, managing large datasets and access requests often requires the employment and training of dedicated staff, and long-term funding for the maintenance of data and biobanks is scarce.^53 55^

Another frequently discussed barrier to equitable data sharing was the scientific environment, where rewards are predominantly grounded in publication but curating and sharing data alone is not grounds for authorship. LMIC stakeholders further reported additional pressures from working multiple roles across teaching and/or medicine, with little time or institutional support for research.^50^ Thus, the burden of data collection is high, while publication outputs are low.^51 61^ Data sharing was seen to exacerbate these existing pressures. LMIC researchers also feared that requirements to publish data before they could perform analyses put them at risk of being scooped by HIC researchers.^31 50 51 62 63^

In some LMIC settings, there is a lack of robust regulatory and governance structures to support equitable data sharing.^58^ Existing ethical frameworks and case studies were thought to primarily address HIC contexts and did not reflect the difficulties LMIC researchers faced.^53^ A lack of enforceable agreements leaves LMIC researchers exposed to exploitation and fails to safeguard participants from harms.^53 64^ The lack of relevant guidance on fair data sharing processes and the ethical dilemmas raised by unfair processes was thought to stagnate action to implement equitable data sharing.^34^

Personal and relational barriers discouraged researchers from sharing data but did not preclude it. Such barriers included a lack of experience with data sharing,^19 21 61^ lack of trust in secondary researchers,^21 50 51 61^ data sovereignty concerns^21 31 47 49 50^ and fear of the imbalance between burdens and rewards created by inequitable data sharing practices (table 2).^21 49 52 61 62^

### Views on foundations for equitable data sharing

The identified literature revealed three key environmental and relational foundations for equitable data sharing: redressing capacity inequities, building trust and creating robust governance and policy structures (table 2). The first included unequal research system capacity between HICs and LMICs across technological and human resources, including expertise in data curation, management and analysis.^31 60^ Significant investment is required to build both technological and human capacity in LMICs to enable equitable data sharing.^31 51 53 63 65^ Dedicated funding for building capacity for data sharing at institutional or national levels, rather than at the project level, resources for sustainable and long-term data sharing (such as funding data access committees and data managers) as well as flexible microfinancing programmes (Microfinancing programs provide very small sums, are easily applied for, and flexible in how they might be spent.) were among the strategies put forward to address this gap.^31 50 51 59^ Building trust occurs through developing long-term relationships and establishing fair and collaborative partnerships between primary and secondary researchers.^19 21 31 34^ Alignment of interests between primary and secondary researchers and accessing institutions that had their own policies supporting equitable data sharing were seen as important factors that built trust.^21 31^

Most authors called for governance mechanisms to be implemented to ensure fair processes and to protect the interests of LMIC researchers and participants.^12 19 21 49 53 55 55 63 64^ They recommended data sharing policies and agreements be enforceable to encourage adherence and to foster trust between primary and secondary researchers.^19 33 34 47 53 61^ Governance structures are required at all levels, from the institutional level to the international level, and are largely lacking in the current data sharing environment.^12 19–21 31 47 49 53 55 57 58 60 63 64^ Community engagement, LMIC researcher engagement, transparency and accountability to external review were considered important in ensuring that governance structures operated ethically and did not perpetuate an exploitative or neocolonial dynamic.^33 58 61 63^

### Views on best practices to promote equitable data sharing

Views about best practices to promote equitable data sharing spanned four themes: advancing recognition and reciprocity (via fair benefit and burden sharing); providing protections for researchers, participants and communities; upholding obligations; and data sovereignty. Data sharing policies and practices should be appropriate to the content and type of data collected, curated and shared. This analysis encompasses the breadth of contexts addressed in the reviewed literature and not all recommendations will be relevant to all types of data sharing.

Benefit sharing with primary researchers was thought to be an important dimension of equitable data sharing.^19 34 47 51 57 61 63^ Most stakeholders called for authorship or another, equal form of academic recognition to balance the burdens of data sharing.^12 21 31 34 47 49 50 52 53 58 59 61 63^ Acknowledgement of primary researchers in publications by secondary researchers was not considered sufficient recognition or reward. Other benefits to be shared with primary researchers included professional promotions, partnerships for mutual exchanges of data, training and skills sharing.^52 53 61^

Benefit sharing with participants or originating communities was also frequently identified as an important aspect of equitable data sharing,^19–21 31 33 47 53 58 60 61 63 64^ as was prioritising secondary research with the potential to translate to local health benefits for originating communities.^19–21 33 61 63^ In recognition of the inequities in access to health resources in originating communities, stakeholders felt that data sharing practices should not contribute to widening these differences, but instead should work to promote global health equity.^33^ Accordingly, it was often felt that secondary research responsive to the needs of originating communities and/or that has translational potential should be prioritised.^19–21 33 61 63^ Developing secondary research agendas in partnership with communities and local health policy-makers was seen as a way to promote research with the potential to benefit communities.^19 53^ Where this was not possible, stakeholders concluded that benefits should at least be shared with originating communities by making the results of secondary analyses available to local researchers, communities and health ministries.^58 60 63^ Some community representatives were supportive of health benefits accruing to the wider public, rather than directly back to their communities.^19^ This was in recognition of the fact that health research data, especially when publicly funded, constitutes a public good. Stakeholders further asserted that commercial outputs of secondary research (such as vaccines) should be made available to originating communities.^60 64^

As secondary researchers benefit from data sharing, some authors felt they should also share the burdens created by such sharing in line with the principle of reciprocity.^12 21 47^ Sharing the cost of managing the dataset was the main form of burden sharing identified. This was seen as particularly relevant in the case of legacy datasets, where costs incurred in the preparation and sharing of data for secondary use are not provided for by the initial funding.^12^

Protections for LMIC stakeholders focused on minimising harms from data sharing. For researchers, these included implementing managed access to data and embargo periods to allow lower-resourced LMIC researchers to publish articles from their data first.^12 19–21 31 33 47 49 51 52 54 61 62^ For participants and communities, minimising harms focused on protecting privacy, preventing stigma and reducing misinterpretation of data during secondary analyses.^19 20 49 52 53 58 60 64^ Proposed means for protecting the community and participant interests were twofold:

1. Placing additional regulations on non-local data-access requests. (We note that some stakeholders preferred to encourage local collaborations to build regional research capacity, while others preferred international collaborations for the opportunity of skills sharing and to enhance their reputation.)^19 20 49 52^
2. Community engagement to determine what constitutes sensitive data, what secondary uses might lead to harm or stigma and what regulations/limitations should be in place for secondary access.^52 53^

Although some stakeholders called for open access to data, many considered this a potentially harmful policy.^20 21 31 33 47 49 61 63 64^ While open access approaches allow for maximum utility and transparency, authors and most stakeholders felt that the potential harms to LMIC researchers and participants (table 1) outweighed this benefit.

Secondary researchers, journals and funders were thought to have obligations to facilitate equitable data sharing processes. In relation to secondary researchers, this included a duty to contribute to building LMIC research capacity.^19 21 31 33 34 47 53 61^ Where possible, secondary researchers were expected to proactively invite primary researchers to collaborate in designing and conducting secondary analyses.^20 47 52 53^ It was acknowledged, however, that collaboration is not always needed nor possible and, in such instances, secondary researchers should make efforts to fairly share benefits with primary researchers in terms of recognition and with originating communities by returning results.^47 63^ Journals were also seen to carry obligations, including making it a condition of publication that results of secondary analyses are disseminated to originating communities, primary researchers, other researchers and ministries of health where the data was collected.^53 63^ Lastly, if funders are to mandate data sharing, it was suggested they should invest in the required technology and human resources to manage the dataset, as well as building analytical capacity, so that LMIC researchers can also perform secondary analyses of shared data.^31 47 52 59 63^

Some models of data sovereignty were thought to advance equitable data sharing, whereas others were thought to obstruct it. A custodianship model places an onus on the custodian to honour their ethical obligations to data subjects as well as to the best interests of research and the broader public, thus balancing the interests of data producers, data subjects and the role of data for public good.^20 31 47 64^ Other stakeholders preferred a model based on ownership and intellectual property rights. Ownership rights were viewed as a foundation for academic recognition and as the basis to restrict access to data if required.^20 21 49^

Collectively, the reviewed literature suggests that building enabling foundations and implementing practices to promote equitable sharing have the potential to address the barriers and concerns raised by LMIC stakeholders (online supplemental file 3). However, none of the enabling foundations combat the environmental barrier presented by the competitiveness of the research environment, where rewards are predominantly grounded in publication.

10.1136/bmjgh-2022-010157.supp3 Supplementary data

## Discussion

This review found that data sharing under existing mandates to share data (with minimal restrictions) risks perpetuating inequities between researchers and countries.^50 61^ Rich data production in LMICs does not necessarily translate to rich rewards, due to the lack of local research capacity and infrastructure.^61^ A lack of adequate protections risks perpetuating a neocolonial dynamic whereby raw data is mined from LMICs by HIC researchers, who then receive the rewards in terms of publications and improved public health in their own countries.^20^ The current data sharing regime, which gives primacy to utility, is thus reinforcing asymmetries in power and privilege and relations of coloniality that are inherent in global health. In recent years, such unfair power dynamics have been the focus of renewed attention in global health research and practice more broadly.^45 66–68^ In such a climate, mandatory data sharing can be inherently punitive to LMIC researchers who must comply with funder requirements or risk losing access to their research support.^61^ Anane-Sarpong further posits that, given the inequities between LMIC and HIC researchers, creating a norm of data sharing without addressing the inequities between LMIC and HIC researchers that is greater than so far laid out in the literature.^61^

The way forward, as proposed in the reviewed literature, is to prioritise equity in data sharing. There was an overarching call for equitable rather than open data sharing.^20 21 31 33 47 49 61 63 64^ Yet, there is a continued lack of understanding and guidance on equitable data sharing. Current data sharing policies and guidelines also often marginalise or do not reflect LMIC voices and fail to address structural inequities in knowledge production landscapes. Moving forward, as a matter of procedural fairness and epistemic justice, we must ensure that any concept of equitable data sharing that is developed ultimately reflects and incorporates the voices of LMIC researchers and communities (as well as other stakeholders).

Published views suggest that LMIC stakeholders believe that, while equitable data sharing should encompass specific practices to implement at the research collaboration/project level, it cannot be achieved without addressing structural inequities within global health research. Equitable data sharing requires fixing the grossly unequal system of global health knowledge production. Key long-term approaches to address structural inequities include: redressing capacity inequities in data curation, data sharing and the analysis of shared data to provide greater opportunities for LMIC researchers to lead secondary analyses and become lead authors on resulting publications; creating robust governance and policy structures at international, national and institutional levels; and building trust, which is consistent with findings of the earlier review by Bull *et al*.^1^ Significant resources are needed to support the collection, curation and effective sharing of high-quality data to advance global health.^69 70^ Beyond these approaches, structural changes are needed to alter how researchers are rewarded in the research environment, including rewards predominantly grounded in peer-reviewed publications.

At the research collaboration/project level, LMIC stakeholders identified key practices to promote equity in data sharing, including appropriate recognition of the work by primary researchers, reciprocity involving fair sharing of benefits and burdens, capacity building collaborations between primary and secondary researchers, and appropriate funding for data sharing activities.^53^ Some of these key practices may also be best supported or made easier by structural changes. For example, adequate recognition could be facilitated by reform of how funders and research institutions evaluate and reward researchers.^71–73^ In this review, LMIC researchers overwhelmingly called for authorship of publications reporting the results of secondary analyses of shared data in order to appropriately recognise the burdens of gathering and maintaining a dataset.^12 21 31 34 47 49 50 52 53 58 59 61 63^ Globally, current promotion criteria and funding for future research remain closely tied to publication in peer-reviewed journals.^31 50 51 61^ Hence, authorship credit is seen as an important element of benefit sharing. Other forms of recognition and acknowledgement in publications were considered insufficient reward for data sharing.

However, we suggest that the needed reform here may not be for journals to alter authorship criteria. Funders and research institutions could incorporate additional metrics beyond quantity, quality and citation impact of publications such as the number of studies using shared data, similar to benchmarks for citations. Such approaches have the potential to directly recognise and reward the sharing of high-quality data,^71 72^ and highlight the responsibilities of multiple stakeholders to promote effective mechanisms for appropriate recognition throughout data sharing cycles.

Building enabling foundations is associated with significant financial costs and can take considerable time. Yet, not pursuing structural change also poses a risk to the utility of research and to public health gains in LMICs: heavy burdens and lack of capacity perpetuates low scientific productivity in the region, hampering the production of new knowledge and slowing progress toward improved health outcomes. In such contexts, onerous data sharing obligations encourage sharing technically unusable data that contributes little or nothing towards reducing health burdens and reduces the utility of shared data.^19 57 59 61 70^ Supporting a longer term effective global health research enterprise and LMIC knowledge production is thus best served by addressing structural inequities and promoting capacity to address local disease burdens via primary and secondary research.

This review was limited to academic peer-reviewed literature and English-language publications. Views from LMICs in South America and the Middle East were lacking. Further exploration is required into views of stakeholders from these locations and from non-English language speakers. The review was also biased by the predominance of contributions to the identified literature being affiliated with the Wellcome Trust, a major funder of global health research. Future research could usefully explore best practices in equitable data sharing in global health research funded by other entities. It could also review and analyse the opinions and conceptions of equitable data sharing expressed by LMIC stakeholders in grey literature and other formats. This review only captured a published, peer-reviewed literature. Going beyond, the peer-reviewed literature is required to ensure LMIC stakeholders’ experiences, perspectives and interests inform the development of equitable data sharing frameworks. Qualitative studies may also be important to capture diverse and less heard viewpoints.

This study did not include LMIC collaborators. Working with them would likely have enriched our analysis. The study was conducted as part of a 3-month student research project to carry out a scoping review of the ethics literature on data sharing from 2015 onwards. The first author chose to focus data analysis on literature within that dataset written by LMIC authors because she wanted to concentrate on less heard voices in data sharing. This occurred mid-way through the project and, at the time, due to a lack of resources to compensate an LMIC collaborator and the project’s design being complete, we felt uncomfortable bringing another collaborator on board. However, an LMIC researcher’s perspective could have further contextualised our analysis and thus provided further insights, including a more nuanced understanding of the structural challenges facing LMIC researchers in relation to equitable data sharing. During analysis and writing up, the second author drew on her experience of conducting research in this field to identify areas where the initial interpretation of findings warranted additional consideration and discussion by all authors, including reflections on positionality.

Ultimately, the study draws attention to the importance of structural change in enabling equitable data sharing. Similarly, there are growing calls for making structural changes in the broader global health enterprise in order to decolonise the field and address unfair power dynamics and epistemic injustices.^74^ As such, it is imperative that future discussions and efforts to make structural changes in global health identify and encompass structural changes that are needed to ensure researchers worldwide are able to generate, curate, share and access data for secondary analyses to address pressing global health problems. To promote epistemic justice and procedural fairness, such work should meaningfully engage relevant data sharing actors, including those who are often marginalised. To achieve equitable data sharing, it must be part of a broader ongoing and inclusive conversation about redressing structural inequities in global health.

A key area for further investigation is mapping out the next steps to obtain a clear conception of equitable data sharing. When it comes to secondary uses of data, there are a number of actors, including LMIC stakeholders, with potentially competing priorities and interests that need to recognised and respected. A conception of equitable data sharing that fairly balances these interests needs to be elucidated in order to advance progress toward equitable data sharing in global health.

## Data Availability

Data are available upon reasonable request. Data are available upon reasonable request from the corresponding author.
