# Supplementary material for: What constitutes equitable data sharing in global health research? A scoping review of the literature on low-income and middle-income country stakeholders’ perspectives
Source: BMJ Glob Health. 2023 Mar 28;8(3):e010157. doi: 10.1136/bmjgh-2022-010157 (PMC10069505; doi:10.1136/bmjgh-2022-010157)
Supplement: Supplementary data [file bmjgh-2022-010157supp001.pdf]

Supplement 1. Final search strategy

Global Health Database

	Searches	Results
**1**	(title:(justice or fairness or fair or ethics or ethical or equit*) OR ab:(justice or fairness or fair or ethics or ethical or equit*) OR ab:(benefit and sharing) AND yr:[2016 TO 2020]) AND (title:(health or medical) OR ab:(health or medical) AND yr:[2016 TO 2020]) AND (((title:((data or database* or dataset*) and (share* or sharing or release* or releasing or disseminat* or distribut* or export* or recycl* or access* or reuse* or management or ownership)) OR title:(information and (share* or sharing or release* or releasing or disseminat* or distribut* or export* or recycl* or access* or reuse* or management or ownership)) OR title:(secondary analysis) OR title:(finding* and (share* or sharing or release* or releasing or disseminat* or distribut* or export* or recycl* or access* or reuse* or management or ownership)) OR title:(result* and (share* or sharing or release* or releasing or disseminat* or distribut* or export* or recycl* or access* or reuse* or management or ownership)) OR title:(data and research))) AND yr:[2016 TO 2020])	68

Embase (Ovid SP) and Medline (Ovid SP)

	Searches	Results
**1**	((data or database* or dataset*) and (share* or sharing or release* or releasing or disseminat* or distribut* or export* or recycl* or access* or reuse* or management or ownership)).ti.	24500
**2**	((information and (share* or sharing or release* or releasing or disseminat* or distribut* or export* or recycl* or access* or reuse* or management or ownership)) or "secondary analysis").ti.	19385
**3**	(finding* and (share* or sharing or release* or releasing or disseminat* or distribut* or export* or recycl* or access* or reuse* or management or ownership)).ti.	5202
**4**	(result* and (share* or sharing or release* or releasing or disseminat* or distribut* or export* or recycl* or access* or reuse* or management or ownership)).ti.	21244
**5**	(data or database or research or science).ti.	1165568
**6**	((data or database* or research or science) and ("open access" or open)).ti,ab.	339103

**7**	(justice or fairness or fair or ethics or ethical or equit* or "benefit sharing").ti,ab.	472661
**8**	1 or 2 or 3 or 4 or 6	405201
**9**	(health or medical).mp.	10488669
**10**	5 and 7 and 8 and 9	1319
**11**	10	1319
**12**	limit 11 to english language	1268
**13**	limit 12 to humans	1035
**14**	limit 13 to yr="2015 - 2021"	616

Web of Science

	**Search**	**Results**
**# 1**	ti= ((data or database* or dataset*)<br><br>and<br><br>(share* or sharing or release* or releasing or disseminat* or distribut* or export* or recycl* or access* or reuse* or management or ownership)<br><br>Indexes=SCI-EXPANDED, SSCI, A&HCI, CPCI-S, CPCI-SSH, BKCI-S, BKCI-SSH, ESCI, CCR-EXPANDED, IC Timespan=All years	53,518
**# 2**	ti=(information and (share* or sharing or release* or releasing or disseminat* or distribut* or export* or recycl* or access* or reuse* or management or ownership) or "secondary analysis")<br><br>Indexes=SCI-EXPANDED, SSCI, A&HCI, CPCI-S, CPCI-SSH, BKCI-S, BKCI-SSH, ESCI, CCR-EXPANDED, IC Timespan=All years	33,586
**# 3**	ti=(finding* and (share* or sharing or release* or releasing or disseminat* or distribut* or export* or recycl* or access* or reuse* or management or ownership)<br><br>Indexes=SCI-EXPANDED, SSCI, A&HCI, CPCI-S, CPCI-SSH, BKCI-S, BKCI-SSH, ESCI, CCR-EXPANDED, IC Timespan=All years	3,611

**# 4**	ti= (result* and (share* or sharing or release* or releasing or disseminat* or distribut* or export* or recycl* or access* or reuse* or management or ownership)<br><br>Indexes=SCI-EXPANDED, SSCI, A&HCI, CPCI-S, CPCI-SSH, BKCI-S, BKCI-SSH, ESCI, CCR-EXPANDED, IC Timespan=All years	15,654
**# 5**	ti= (data or database or research or science)<br><br>Indexes=SCI-EXPANDED, SSCI, A&HCI, CPCI-S, CPCI-SSH, BKCI-S, BKCI-SSH, ESCI, CCR-EXPANDED, IC Timespan=All years	1,797,781
**# 6**	ts= ((data or database* or research or science)<br><br>and ("open access" or open)<br><br>Indexes=SCI-EXPANDED, SSCI, A&HCI, CPCI-S, CPCI-SSH, BKCI-S, BKCI-SSH, ESCI, CCR-EXPANDED, IC Timespan=All years	426,849
**# 7**	ts=(justice<br>or fairness or fair or ethics or ethical or equit* or "benefit<br><br>sharing")<br><br>Indexes=SCI-EXPANDED, SSCI, A&HCI, CPCI-S, CPCI-SSH, BKCI-S, BKCI-SSH, ESCI, CCR-EXPANDED, IC Timespan=All years	571,310
**# 8**	#1 or #2 or #3 or #4 or #6<br><br>Indexes=SCI-EXPANDED, SSCI, A&HCI, CPCI-S, CPCI-SSH, BKCI-S, BKCI-SSH, ESCI, CCR-EXPANDED, IC Timespan=All years	526,569
**# 9**	ts= (health or medical)<br><br>Indexes=SCI-EXPANDED, SSCI, A&HCI, CPCI-S, CPCI-SSH, BKCI-S, BKCI-SSH, ESCI, CCR-EXPANDED, IC Timespan=All years	3,256,404
**# 10**	#5 and #7 and #8 and #9<br><br>Indexes=SCI-EXPANDED, SSCI, A&HCI, CPCI-S, CPCI-SSH, BKCI-S, BKCI-SSH, ESCI, CCR-EXPANDED, IC Timespan=All years	711
**# 11**	(#10) *AND* LANGUAGE: (English)<br><br>Indexes=SCI-EXPANDED, SSCI, A&HCI, CPCI-S, CPCI-SSH, BKCI-S, BKCI-SSH, ESCI, CCR-EXPANDED, IC Timespan=2016-2020	417

**#12**	(#10) *AND* LANGUAGE: (English)<br><br>Refined by: [excluding] WEB OF SCIENCE CATEGORIES: (LINGUISTICS OR MATHEMATICAL COMPUTATIONAL BIOLOGY OR ENVIRONMENTAL SCIENCES OR CRIMINOLOGY PENOLOGY OR BIODIVERSITY CONSERVATION OR CELL TISSUE ENGINEERING OR REGIONAL URBAN PLANNING OR CHEMISTRY ANALYTICAL OR TELECOMMUNICATIONS OR CHEMISTRY APPLIED OR TRANSPORTATION OR URBAN STUDIES OR ENGINEERING INDUSTRIAL OR ENVIRONMENTAL STUDIES OR GEOGRAPHY PHYSICAL OR ENGINEERING ELECTRICAL ELECTRONIC OR ENGINEERING MULTIDISCIPLINARY OR GREEN SUSTAINABLE SCIENCE TECHNOLOGY)<br><br>Timespan: 2016-2020. Indexes: SCI-EXPANDED, SSCI, A&HCI, CPCI-S, CPCI-SSH, BKCI-S, BKCI-SSH, ESCI, CCR-EXPANDED, IC.	384
