# Supplementary material for: What constitutes equitable data sharing in global health research? A scoping review of the literature on low-income and middle-income country stakeholders’ perspectives
Source: BMJ Glob Health. 2023 Mar 28;8(3):e010157. doi: 10.1136/bmjgh-2022-010157 (PMC10069505; doi:10.1136/bmjgh-2022-010157)
Supplement: Supplementary data [file bmjgh-2022-010157supp002.pdf]

Supplement 2. Articles included in the review

Year	Author	Article type	Location of institutional affiliation: first author	Location of institutional affiliation: last author
**2015**	Cheah PY, Tangseefa D, Somsaman A, Chunsuttiwat T, Nosten F, Day NPJ, Bull S, Parker M.	Qualitative study	United Kingdom/Thailand	United Kingdom
**2015**	Denny SG, Silaigwana B, Wassenaar D, Bull S, Parker M.	Qualitative study	South Africa	United Kingdom
**2015**	Hate K, Meherally S, More NS, Jayaraman A, Bull S, Parker M, Osrin D.	Qualitative study	India	United Kingdom
**2015**	Jao I, Kombe F, Mwalukore S, Bull S, Parker M, Kamuya D, Molyneux S, Marsh V.(a)	Qualitative study	Kenya	Kenya/United Kingdom
**2015**	Jao I, Kombe F, Mwalukore S, Bull S, Parker M, Kamuya D, Molyneux S, Marsh V.(b)	Qualitative study	Kenya	Kenya/United Kingdom
**2015**	Merson L, Phong TV, Nhan LNT, Dung NT, Ngan TTD, Kinh NV, Parker M, Bull S..	Qualitative study	United Kingdom/Vietnam	United Kingdom

**2016**	Hayes R, Ayles H, Binka F, Cowan F, Kamali A, Kapiga S, Kleinschmidt I, Mayaud P, Patel V, Smith P, Weiss H.	Commentary/Conceptual analysis	United Kingdom	United Kingdom[1]
**2016**	Merson L, Gaye O, Guerin PJ	Commentary/Conceptual analysis	United Kingdom/Vietnam	United Kingdom
**2016**	Rappert B, Bezuidenhout L	Qualitative study	United Kingdom	United Kingdom/South Africa
**2017**	Bezuidenhout L, Kelly AH, Leonelli S, Rappert B	Qualitative study	United Kingdom/South Africa	United Kingdom
**2017**	Cheah PY, Day NPJ	Case study	United Kingdom/Thailand	Thailand/United Kingdom
**2017**	Cheah PY, Day NPJ, Parker M, Bull S	Case study	United Kingdom/Thailand	United Kingdom
**2018**	Akintola SO. (2018)	Commentary/Conceptual analysis	Nigeria	n/a
**2018**	Anane-Sarpong E, Wangmo T, Ward CL, Sankoh O, Tanner M, Elger BS.	Qualitative study	Ghana/Switzerland	Switzerland
**2018**	Bezuidenhout L, Chakauya E	Quantitative study	United Kingdom/South Africa	South Africa
**2018**	Serwadda D, Ndebele P, Grabowski MK, Bajunirwe F, Wanyenze RK	Commentary/Conceptual analysis	Uganda	Uganda

[1] These authors were affiliated with the London School of Hygiene & Tropical Medicine, and are embedded in projects in Uganda, Tanzania and India

**2019**	Andanda P	Commentary/Conceptual analysis	South Africa	n/a
**2019**	Barnes KI, Canario JA, Vernekar SS, Goudar SS, Espinal R, Merson L, Cheah PY.	Case study	South Africa	United Kingdom/Thailand
**2019**	Fernando B, King M, Sumathipala A	Case study	Sri Lanka	United Kingdom/Sri Lanka
**2019**	Humphreys GS, Tinto H, Barnes KI.	Case study	United Kingdom	South Africa
**2019**	Vaz M, Palmero AG, Nyangulu W, Diallo AA, Ho CWL	Commentary/Conceptual analysis	India	Singapore/Hong Kong
**2019**	Waithira N, Mutinda B, Cheah PY	Commentary/Conceptual analysis	Thailand/United Kingdom	Thailand/United Kingdom
**2020**	Anane-Sarpong E, Wangmo T, Tanner M.	Commentary/Conceptual analysis	Ghana/Switzerland	Switzerland
**2020**	Bull S, Bhagwandin N	Editorial	United Kingdom	South Africa
**2020**	Gorina Y, Redd JT, Hersey S, Jambai A, Meyer P, Kamara AS, Kamara A, Harding JD, Bangura B, Kamara MAM.	Case study	United States of America	Sierra Leone

**2020**	Kaewkungwal J, Adams P, Sattabongkot J, Lie RK, Wendler D.	Qualitative interviews	Thailand	United States of America
