# Supplementary material for: What constitutes equitable data sharing in global health research? A scoping review of the literature on low-income and middle-income country stakeholders’ perspectives
Source: BMJ Glob Health. 2023 Mar 28;8(3):e010157. doi: 10.1136/bmjgh-2022-010157 (PMC10069505; doi:10.1136/bmjgh-2022-010157)
Supplement: Supplementary data [file bmjgh-2022-010157supp003.pdf]

**Supplement 3.** Summary of how barriers to equitable data-sharing and potential threats of unfair data-sharing are addressed by foundations of and best practices in equitable data-sharing

| *Barriers to equitable data-sharing and potential threats of unfair data-sharing* | | *Foundations of equitable data-sharing* | *Practices to promote equitable data-sharing* |
|---|---|---|---|
| *Structural barriers* | • Unequal research systems, resources and capacity between HICs and LMICs leads to greater capacity for HIC researchers to perform timely secondary analysis, whereas LMIC researchers have inadequate funding and expertise to curate, share and maintain data sets | • Capacity building of LMIC research systems; provision of resources for sustainable and long-term data-sharing | • Funders to provide funding for sustainable data-sharing |
| | • Lack of fiscal resources limits capacity to undertake original research | • Capacity building of LMIC research systems; provision of resources for sustainable and long-term data-sharing | • Funders to provide funding for sustainable data-sharing |
| | | • Establish frameworks and policies that support equitable | |

| | | |
|---|---|---|
| • Limited local and national policy and regulatory frameworks to govern data-sharing | data-sharing, backed by enforceable governance mechanisms<br>• Accessing institutions should have their own policies supporting equitable data-sharing | • Inclusion of LMIC stakeholders at all levels, including communities in developing data-sharing policies |
| • Lack of enforceability of data-sharing agreements and policies | • Establish frameworks and policies that support equitable data-sharing, backed by enforceable governance mechanisms | • Enforceable data-sharing agreements between primary researchers and secondary researchers |
| • Competitive scientific research environment, and rewards grounded in publication | • Fair rewards for high-quality data collection, curation and sharing | • Secondary user duties: offer collaboration with primary researchers where possible and appropriate; so that rewards and knowledge are shared with primary researchers<br>• Benefit-sharing and reciprocity: authorship opportunities or other forms of academic recognition on par with the burdens of data-sharing |

| | | | |
|---|---|---|---|
| *Potential threats to researchers posed by unfair data-sharing* | • Worsening of unfair power relations with HIC researchers | • Capacity building of LMIC research systems; provision of resources for sustainable and long-term data-sharing<br>• Establish frameworks and policies that support equitable data-sharing, backed by enforceable governance mechanisms<br>• Equitable partnerships with secondary researchers | • Inclusion of LMIC stakeholders at all levels, including communities in developing data-sharing policies<br>• Benefit-sharing and reciprocity: authorship opportunities or other forms of academic recognition on par with the burdens of data-sharing; secondary-analysis should have local translational benefits where possible; allow community input into research agendas<br>• Burden-sharing: the cost of managing the data should be shared with secondary researchers<br>• Secondary researcher duties: contribute to building local research capacity; offer collaboration with primary researchers |
| | • Opportunity and fiscal cost unfairly burden poorly resourced researchers and institutions; and the rewards are insufficient to offset the burdens | • Capacity building of LMIC research systems; provision of resources for sustainable and long-term data-sharing | • Funders to provide funding for sustainable data-sharing |

| | | |
|---|---|---|
| • Exclusion from participation in advancing science (LMIC researchers are stuck in a cycle of data production only) | • Accessing institutions should have their own policies supporting equitable data-sharing<br>• Equitable partnerships with secondary researchers<br>• Capacity building of LMIC research systems; provision of resources for sustainable and long-term data-sharing | • Funders to provide funding for sustainable data-sharing<br>• Secondary user duties: offer collaboration with primary researchers |
| • Career and reputational damage leading to loss of funding opportunities | • Alignment of interests between primary and secondary researchers<br>• Equitable partnerships with secondary researchers | • N/A |
| • Lack of recognition hampers career progression | • Equitable partnerships with secondary researchers | • Benefit-sharing and reciprocity: authorship opportunities or other forms of academic recognition on par with the burdens of data-sharing; secondary-analysis should have local translational benefits where possible; allow community input into research agendas |

| | | | |
|---|---|---|---|
| *Potential threats to participants and communities posed by unfair data-sharing* | • Commercial exploitation of participants and communities | • Establish frameworks and policies that support equitable data-sharing, backed by enforceable governance mechanisms | • Restricted/managed access<br>• Benefit-sharing and reciprocity secondary-analysis should have local translational benefits where possible; allow community input into research agendas |
| | • Stigmatisation | • Equitable collaborations between primary and secondary researchers – reduces the risk of reporting results in a way that increases stigmatisation of participants and communities | • Inclusion of LMIC stakeholders at all levels, including communities in developing data-sharing policies<br>• Allow community input into research agendas |
| | • Benefits of research are not shared with originating communities | • Establish frameworks and policies that support equitable data-sharing, backed by enforceable governance mechanisms | • Secondary analysis should have local translational benefits where possible<br>• Accountability: feedback of results of secondary analysis to community representatives |

| | | | |
|---|---|---|---|
| *Potential threats to local health systems posed by unfair data-sharing* | • Reduced opportunity to develop local research infrastructure and personnel | • Capacity building of LMIC research systems; provision of resources for sustainable and long-term data-sharing | • Secondary researcher duties: contribute to building local research capacity<br>• Funders to provide funding for sustainable data-sharing |
